# A novel c.116 - 117 del variant in Unverricht-Lundborg disease: first ULD report in large Chinese population and review of the pathogenetic variants in *CSTB* gene

**DOI:** 10.1186/s42494-025-00216-4

**Published:** 2025-05-29

**Authors:** Pu Miao, Yao Ding, Zhidong Cen, Yulan Chen, Wei Luo, Baorong Zhang, Zhiying Wu, Meiping Ding, Shuang Wang

**Affiliations:** 1 Department of Pediatric, Second Affiliated Hospital, School of Medicine, Zhejiang University, Hangzhou, 310009 China; 2 Department of Neurology, Epilepsy Center, Second Affiliated Hospital, School of Medicine, Zhejiang University, Hangzhou, 310009 China

**Keywords:** Unverricht-Lundborg disease, *CSTB* gene, Perampanel

## Abstract

**Background:**

Unverricht-Lundborg disease (ULD) is a rare autosomal recessive neurodegenerative disorder, often caused by biallelic promoter expansions of *CSTB* gene or, more rarely by point/indel variants. The best-known area for ULD are the shores of the Baltic and Mediterranean Sea and few cases have been recorded from Asia.

**Case presentation:**

In this report, we present the first case of a Chinese patient with ULD. The patient was a 21-year-old female with normal cognitive function. She developed nocturnal bilateral tonic–clonic seizures (BTCS) at age 8, with subsequent onset of myoclonic jerks along with ataxia at age 12. Myoclonic jerks were triggered by flashing lights and during menstrual periods. EEG recording showed multifocal spikes and sharp-waves, predominantly in bilateral occipital regions. Genetic testing revealed heterozygous compound variants for a novel indel variant (c.116 - 117 delAG) and the repeat expansion of *CSTB* gene. The refractory BTCS and myoclonic jerks showed remarkable response to low-dose (2 mg/day) perampanel treatment. After 24 months of follow-up, the patient remained seizure-free, but her myoclonic jerks recurred, which could be reduced by increasing the dosage of perampanel.

**Conclusions:**

To the best of our knowledge, this is the first report of ULD in the large Chinese population. By comparison with homozygous promoter expansions, we found an earlier age of first symptom onset and more refractory BTCS of ULD patients with compound or homozygous point/indel variants.

## Background

Unverricht-Lundborg disease (ULD) is an autosomal recessive neurodegenerative disorder caused by biallelic alterations of the *CSTB* gene. ULD is characterized by bilateral tonic–clonic seizures (BTCS), myoclonus, mild cognitive decline, and other neurological symptoms such as ataxia, dysarthria, and psychiatric disorders [1]. The most frequent genetic cause of ULD is the unstable expansion (> 30 times) of a dodecamer repeat expansion. In rare cases it can also occur in compound heterozygous (a dodecamer repeat expansion and a sequence variant such as single-nucleotide variant or indel), or even in homozygous *CSTB* point or indel variants [2, 3]. To date, less than 20 pathogenic *CSTB* disease-causing variants have been reported in ULD patients [4]. Therefore, profound relationship between genotype and phenotype has not been fully established.

ULD is most reported in Finland, where the total ULD population is estimated to be only around two hundreds [5]. While sporadic cases have been reported globally, including in Asian countries such as Japan and Korea, no cases of ULD have been documented in the Chinese population, despite it being the world's largest ethnic group, with over 1.4 billion people worldwide. It is worth noting that about 10% of ULD patients exhibit a mild phenotype, which could result in underdiagnosis [5]. However, it could not explain why such a large population has never been documented to have ULD patients.

Here, we report a case of ULD with a definite genetic diagnosis in a Chinese patient. Our patient was found to have a compound heterozygous variant of a novel indel (c.116 - 117 delAG) and a dodecamer repeat expansion in the *CSTB* gene, confirming the existence of the pathogenic genetic background of *CSTB* gene in the Chinese population. We also reviewed and compared the phenotype of patients with different genetic types of *CSTB* gene.

## Case presentation

The patient was a 21-year-old female, graduating from the junior high school. She was born at term by non-consanguineous parents and her psychomotor development was normal. She reported no family history of epilepsy or other neurological disorders. Her first bilateral tonic–clonic seizure (BTCS) occurred when she was 8 years old and was triggered by bright lights. She subsequently experienced recurrent nocturnal convulsive seizures. Initially, she was treated with oxcarbazepine, which proved ineffective. At the age of 12, she developed myoclonic jerks that were usually triggered by flashing lights and menstrual periods. Valproate was introduced, which reduced the frequency of myoclonic jerks. However, her symptoms worsened at the age of 18, and both valproate and levetiracetam showed a poor response. She experienced 1–2 BTCSs per month (all occurring at night), 2–3 myoclonic seizures per week, and myoclonic jerks triggered by light or when she was in a state of tension.

**Fig. 1:**
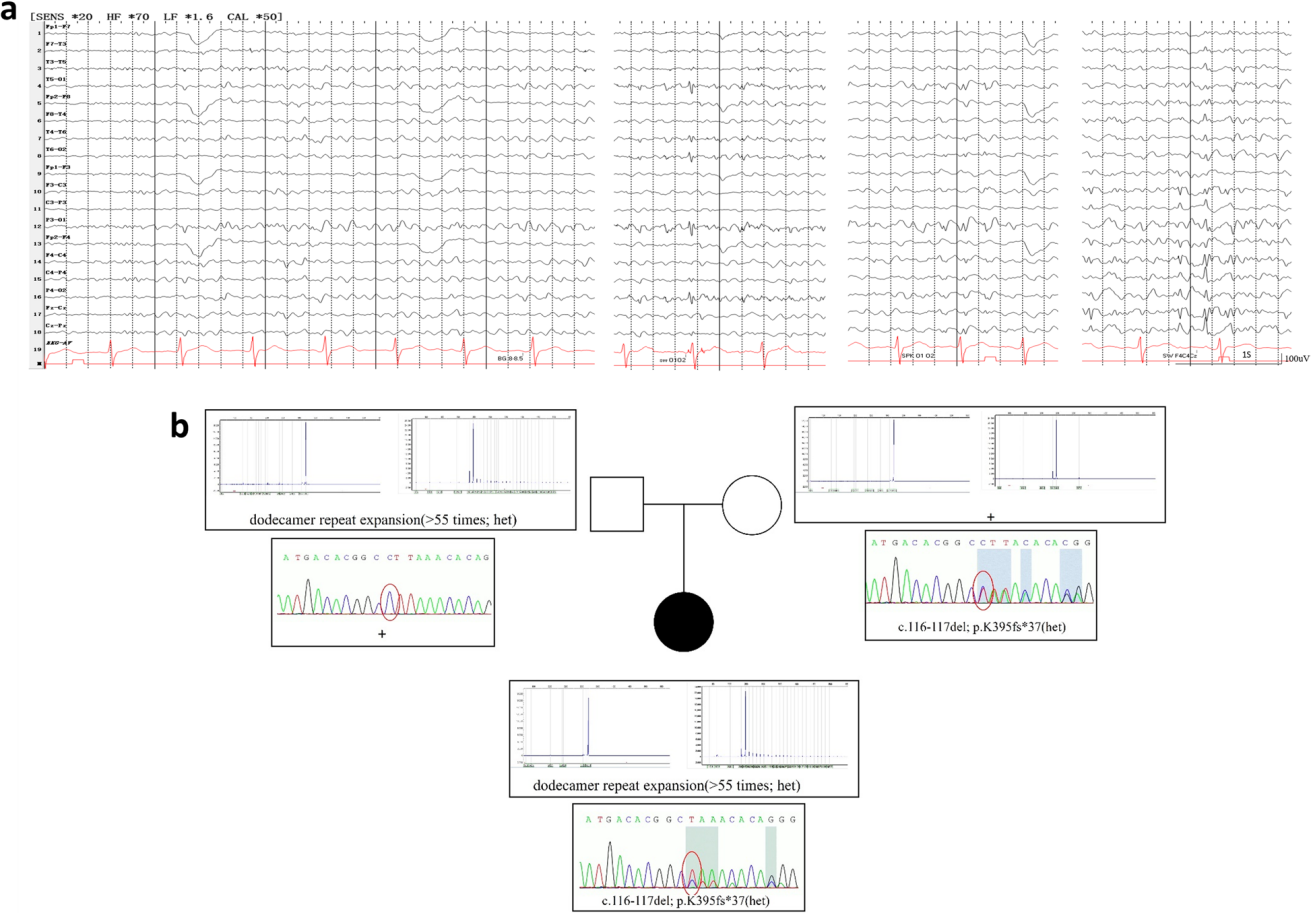
**a** EEG recording showed normal background and multifocal spike-wave, sharp-wave epileptiform discharges, predominantly in bilateral occipital regions (O_1_, O_2_ electrodes). **b** Genetic findings of the patient. Pedigree of the family and PCR amplification-capillary electrophoresis and sanger sequencing showing the segregation of the variants in the family members

The patient was referred to our hospital for preoperative evaluation of refractory epilepsy at the age of 19. The neurological examination revealed no abnormalities except for truncal ataxia. Her brain MRI scan was normal. FDG-PET revealed hypometabolism especially affecting the bilateral frontal-parietal junction regions. Video-EEG recordings showed multifocal spike-wave and sharp-wave epileptiform discharges, predominantly in bilateral occipital regions (electrodes O_1_ and O_2_, Fig. 1a), with normal posterior background during interictal periods. After perampanel was introduced at 2 mg daily, she had a marked improvement in myoclonic jerks and gait. Epileptic seizures, including BTCS and myoclonic seizures, stopped with the use of perampanel (PER). During the two-year follow-up, she remained seizure-free but experienced recurrent symptoms of myoclonic jerks, which responded to PER dose escalation (up to 6 mg/day). No psychological or behavioral side-effects of PER were observed during the follow-up. The patient is currently taking perampanel 6 mg/day and levetiracetam 1000 mg/day.

A PCR-amplification test of the *CSTB* gene identified a pathogenic heterozygous dodecamer repeat expansion (≥ 55 times). Combining this with the clinical phenotype, it supported a diagnosis of ULD disease and necessitated the identification of another variant. Whole-exome sequencing revealed a previously unpublished heterozygous variant (c.116 - 117 del, p.K395fs* 37) in the *CSTB* gene (Fig. 1b). According to the American College of Medical Genetics and Genomics (ACMG) guidelines, it could be evaluated as a likely pathogenic variant (PVS1 + PM2). Further verification confirmed it as a compound heterozygosity.

## Discussion

We have presented the first genetic confirmed case of ULD in the Chinese population, identified through mutations the *CSTB* gene. Our patient had a ten-year misdiagnosis, highlighting the under-reporting of ULD in China. ULD, a form of progressive myoclonic epilepsy (PME), can be difficult to distinguish from juvenile myoclonic epilepsy (JME) and familial cortical myoclonic tremor with epilepsy (FCMTE) in the early stages of the disease. However, the active myoclonus features and the responses to antiseizure medications (ASMs) can be helpful for early identification. Conventional PCR may not be effective in detecting the size of fully expanded alleles and GC-rich sequences in the dodecamer repeat region, which likely contributes to the underdiagnosis of ULD in China. Additionally, the insufficient application of this special genetic testing in China further compounds this issue.

Another noteworthy aspect of ULD is the genotype–phenotype correlation. The *CSTB* gene, the primary genetic causes of ULD, is situated on chromosome 21 at the 21q22.3 locus. This relatively small gene comprises three exons and spans approximately 3 kilobases (kb) of genomic DNA [6]. It encodes the Cystatin B protein, a member of the cysteine protease inhibitor family, which is also referred to as Stefin B. The *CSTB* gene is widely expressed in neural cells and plays a role in brain development such as cell proliferation differentiation and neuronal migration [7]. The *CSTB* gene is the primary genetic cause of ULD and includes three pathogenic types of variants: 1) homozygous promoter expansions in the *CSTB* gene (observed in approximately 80–90% of probands), 2) compound heterozygosity for a *CSTB* dodecamer repeat expansion and a *CSTB* sequence variant, and 3) homozygosity for a *CSTB* sequence variant. In our case, compound heterozygosity with a novel variant, c.116 - 117 delAG (deletion of two nucleotides in exon 2), was found. This variant is predicted to result in a truncated CSTB protein due to a frameshift (p.K39SfsX37), which might lead to a loss of function of the CSTB protein. To date, 13 pathogenic variants in 24 patients, including descriptions of the phenotype, have been reported in association with ULD. By comparing with a group of ULD patients with homozygous promoter expansions in the *CSTB* gene (mean age of first seizures:10.0 ± 2.28 years, range: 5–13 years old, *n* = 16) [1], patients with compound heterozygosity or homozygosity for a *CSTB* sequence variant (mean age of first seizures: 7.5 ± 3.00 years, range: 0.5–13 years, *n* = 16) presented with an earlier age of onset (Mann–Whitney test, *P* = 0.013), as previously reported by Canafoglia et al. [2]. An earlier onset has been associated with a worse prognosis; therefore, the disease course in patients with a pathogenic *CSTB* sequence variant may be less favorable than in patients with common homozygous promoter expansions.

In the majority of cases in Table 1 (78.5%, 11/14), BTCS was recorded as the first symptom, and this percentage was higher than in patients with homozygous promoter expansions [8]. It has been reported that BTCS has a positive response to antiseizure medications (ASMs) and may disappear as the disease progresses. However, our patient suffered from BTCS for nearly ten years and had a poor response to escalating dose of valproic acid (VPA) and levetiracetam (LEV). Similar findings were reported by Koskenkorva et al. among patients with the c.202 C > T variant [7]. None of the patients in Table 1 were on monotherapy, and a few patients even required more than four ASMs. BTCS among patients with compound heterozygosity or homozygosity for a CSTB sequence variant might become refractory to ASMs more easily than in patients with homozygous promoter expansions. The earliest and most used medication was VPA, but nearly all patients had a poor response. Our patient suffered from a long-standing nocturnal BTCS, and a novel medication, PER, that selectively inhibits postsynaptic AMPA receptors, demonstrated a remarkable effect. Although an abatement of PER efficiency for her myoclonic jerks was observed, her BTCS remained well-controlled during the two-year follow-up. PER may be recommended as an effective early treatment option for these patients, especially those with refractory BTCS.

**Table 1:** Description of main clinical and genetic features in ULD patients harboring point or indel disease causing variants of the *CSTB* gene

Num	Y/G	Age at onset	Seizures (year)	Treatment	Cognitive impairment	Ataxia	Mood disorder/walking ability	EEG	Brain MRI	*CSTB* variants NM_000100.4	Types	Descent
1 [8]	/	6–16	BTCS often presenting as the first symptom; stimulus-sensitive myoclonus					SW-PSW	/	218_219 delTC, C	Frame shift	France
2 [8]										169 - 2 A > G, C	Splice site	France
3 [8]										10G > C, H	Missense	Morocco
4 [3]	18, F	0.5	BTCS (0.5)	/	Severe	Yes	None/Wheelchair-bound	SW+	Cerebral atrophy	218 dupT, H	Frame shift	Sri Lankan
5 [3]	10, F	None	None	None	Severe	Yes	None/Wheelchair-bound	Diffuse slowing	Cerebral atrophy			Sri Lankan
6 [9]	33, M	14	BTCS (14), M(18)	PHT, VPA, CZP, LEV, ZNS+VNS	Normal	Yes	None/Normal	SW-PSW; IPS-M	Normal	c.66G > A, H	Splice site	Portugal
7 [10]	> 19, M	/	Myoclonic (/)	/	/	Yes	/	/	Abnormal	c.10G > T, H	Missense	Eastern
8 [2]	42, M	7	M (7), BTCS (10)	VPA, LEV, ZNS, ESM, CZP	Moderate	Yes	Yes/-	SW; IPS-M	Normal	c.67 - 1G > C, C	Splice site	Italian
9 [2]	35, M	9	BTCS (9), M (15)	VPA, LEV, CZP, PRM	Moderate	Yes	None/-	SW+	Normal	c.67 - 1G > C, C	Splice site	Spanish
10 [2]	34, M	5	BTCS (5), M(7)	VPA, LEV, ESM, CZP	Severe	Yes	None/-	SW-PSW; IPS-M	Cerebral atrophy	c.67 - 1G > C, C	Splice site	Spanish
11 [2]	30, M	6	BTCS (6), M(16)	VPA, LEV, LTG, CZP	Normal	Yes	None/-	SW+;IPS-M	Normal	c.168 + 1_18 del, C	Frame shift	Italian
12 [2]	23, M	9	BTCS (9), M(12)	VPA, LEV, LTG, DZP	Mild	None	None/-	PSW	Normal	c.133 C > T, C	Missense	Italian
13 [2]	15, M	6	BTCS (6), M(8)	ZSM, VPA, CZP	Mild	Yes	Yes/-	SW-PSW; IPS-M	Abnormal	c.168 + 2–168 + 21 delinsA, C	Splice site	Italian
14 [2]	16, M	7	BTCS (7), M (9)	ZNS, VPA, LEV, CZP, PIR	Mild	Yes	Yes/-	SW-PSW; IPS-M	Normal			Italian
15 [2]	12, M	10	BTCS (10), M (10)	LEV, PER	Normal	None	None/-	SW-PSW; IPS-M	Normal			Italian
16 [11]	18, M	8	M (8), BTCS(13)	VPA, CBZ, CZP, ZNP, PIR	Mild	None	None/-	SW; IPS-M	Normal	c.168G > A, C	Splice site	Japanese
17 [12]	49, M	8	M (8), BTCS (13)	VPA, LEV, PIR, TPM, CZP	Mild	Yes	Yes/-	SW+	Normal	c.132_134 delGAG, C	In frame	Roman
18 [12]	53, F	13	BTCS (13), M(13)	VPA, PHT	Normal	None	Yes/-	SW	Normal			Roman
19 [7]	23, M	5	BTCS, M	VPA, LTG, LEV, PIR	Severe	None	None/Limited walking ability	SW+; IPS-M	Cerebral atrophy	c.202 C > T, C	Nonsense	Finnish
20 [7]	21, M	7	BTCS,M	VPA, LTG, LEV	Mild	None		SW+; IPS-M	Normal			Finnish
21 [7]	34, F	10	BTCS, M	VPA, CZP, TPM, PIR	Moderate	None	None/Wheelchair bound	SW+; IPS-M	Cerebellar atrophy			Finnish
22 [7]	37, M	7	BTCS, M	VPA, CZP, LEV, PIR	Moderate	None		SW+; IPS-M	Brain atrophy			Finnish
23 [7]	14, F	6	BTCS, M	VPA, CZP	Severe	Yes	None/Normal	SW+; IPS-M	Normal			Finnish
26	21, F	8	BTCS (8),M (12)	OXC, VPA, LEV, PER	Normal	Yes	Yes/Normal	SW; IPS-M	Normal	c.116 - 117 del, C	Deletion	Chinese

*F* Female, *M* Male, *BTCS* Bilateral tonic–clonic seizure, *M* Myoclonous, *SW* Spike weave, *SW+* Spike weave with diffuse background slowing, *IPS-M* IPS-induced myoclonus, *CH* Compound heterozygous, *Hom* Homozygous, *VPA* Valproate, *LEV* Levetiracetam, *ZNS* Zonisamide, *ESM* Ethosuximide, *CZP* Clonazepam, *LTG* Lamotrigine, *PIR* Piracetam, *PER* Perampanel, *CBZ* Carbamazepine, *TPM* Topiramate, *PHT* Phenytoin

In addition, only a fraction of the pathogenic variants are missense (3/13, 23%). Interestingly, we found that patients with missense variants might exhibit a milder phenotype. It has been reported that homozygous promoter expansions leads to a reduced expression of the *CSTB* gene, and lower levels of CSTB expression are associated with a more severe phenotype [3, 4]. Variants affecting splice sites or predicting truncated protein may have a greater impact on CSTB expression than missense variants, which could potentially explain the varying degrees of phenotype severity. Further investigations should focus on understanding the mechanism by which reduced CSTB expression leads to distinct phenotypes.

## Conclusions

In conclusion, we report the first case of ULD in the Chinese population with an unpublished variant c.116 - 117 del in the *CSTB* gene. When combined with other reported compound or homozygous variants, patients with this variant manifested an earlier onset and more refractory BTCS than those with homozygous promoter expansions in the *CSTB* gene. Among ULD patients with compound or homozygous variants, missense variants account for only a small percentage and their phenotype may be milder.

## Data Availability

There were no supporting data.

## Abbreviations

ACMG: American College of Medical Genetics and Genomics
ASMs: Antiseizure medications
BTCS: Bilateral tonic–clonic seizures
FCMTE: Familial cortical myoclonic tremor with epilepsy
JME: Juvenile myoclonic epilepsy
LEV: Levetiracetam
PER: Perampanel
ULD: Unverricht-Lundborg disease
VPA: Valproic acid
